# Change in cascade of care among individuals with hypertension – India hypertension control initiative, 2018-2024

**DOI:** 10.1016/j.ajpc.2026.101705

**Published:** 2026-06-17

**Authors:** Parasuraman Ganeshkumar, Maanasa Rajagopalan, Mosoniro Kriina, Jayashree Somasundaram, Jayashree Kalva, Navaneeth S Krishna, Kannamkottapilly Chandrasekharan Prajitha, Sonie Sait, Vettrichelvan Venkatasamy, Santhiya Venkatesan, Sugavathy A, Sailaja Bitragunta, Meenakshi Sharma, Roopa Shivashankar, Pragati Pragya, Sampada D Bangar, Tapas Chakma, Abhishek Kunwar, Amol Wankhede, Rupali Bharadwaj, Vishwajit Bharadwaj, Sravan Kumar Reddy T, Navneet Kishore, Bidisha Das, Yannick Puthussery, Deenadayalan Chandran, Branch Immanuel A, Sandeep S Gill, Ashu Gupta, Bipin Gopal, Ramakrishna Kodirekkala, Jagannath Reddy M, Sarita Hazare, Namita Neelkanth, Gagandeep Singh Grover, Anupam Khungar Pathni, Prabhdeep Kaur

**Affiliations:** a ICMR, National Institute of Epidemiology, Chennai, India; b Division of Noncommunicable Diseases, Indian Council of Medical Research, New Delhi, India; c Division of Epidemiology and Biostatistics, ICMR-National Institute of Translational Virology and AIDS Research, Pune, India; d Division of Noncommunicable Diseases, ICMR-National Institute of Research In Tribal Health, Jabalpur, India; e Department of Noncommunicable Diseases, WHO Country Office for India, New Delhi, India; f IHCI Project, WHO Country Office for India, New Delhi, India; g IHCI Project, District NCD Cell, Chhindwara Madhya Pradesh, WHO, India; h IHCI Project, District NCD Cell, Wardha Maharashtra, WHO, India; i IHCI Project, State NCD Cell, Hyderabad Telangana, WHO, India; j IHCI Project, State NCD Cell, Chandigarh Punjab, WHO, India; k IHCI Project, District NCD Cell, Bhatinda Punjab, WHO, India; l IHCI Project, District NCD Cell, Thrissur Kerala, WHO, India; m IHCI Project, District NCD Cell, Kannur Kerala, WHO, India; n IHCI Project, District NCD Cell, Thiruvananthapuram Kerala, WHO, India; o State NCD Cell, Department of Health and Family Welfare, Government of Punjab, Chandigarh, India; p State NCD Cell, Directorate of Health Services, Government of Kerala, Thiruvananthapuram, India; q Department of Health and Family Welfare Commissioner, Government of Telangana, Hyderabad, India; r Joint Deputy Director, State NCD Cell, Directorate of Health Services, Government of Maharashtra, Mumbai, India; s State NCD Cell, Ministry of Health and Family Welfare, Directorate of Health Services, India; t IHCI Project, Resolve to Save Lives, New Delhi, India; u Isaac Centre for Public Health, Indian Institute of Science, Bangalore, India

**Keywords:** Cascade of care, Hypertension, Community survey, India hypertension control initiative

## Abstract

**Background:**

Hypertension is one of the major contributors to cardiovascular mortality globally. Periodic hypertension cascade-of-care surveys are important for the health system's planning and monitoring. We conducted a baseline survey (2018), followed by a repeat cross-sectional survey (2023–24) in nine Phase I India Hypertension Control Initiative (IHCI) districts to assess changes in hypertension control.

**Methods:**

We conducted a repeat cross-sectional survey in nine Phase-I IHCI districts across five states: Punjab, Madhya Pradesh, Maharashtra, Telangana, and Kerala. We included adults aged 18 to 69 years using a multi-stage cluster sampling approach, with a sample size of 624 per district. We estimated the proportions with 95% confidence intervals for hypertension awareness, treatment coverage, and control for each district. We analysed the change in the cascade of care for hypertension between the two surveys.

**Results:**

In 2023–24, 7324 individuals had hypertension; 58.5% were aware of their condition, 44.7% were under treatment, and 21.2% had it under control. Compared with 2018, there was an absolute improvement of 6.1%, 3.9%, and 6.7% in hypertension awareness, treatment, and control, respectively. Nearly 70% of individuals aware of their diagnosis were undergoing treatment. The elderly age group (over 60 years) showed greater improvement across all three hypertension cascade indicators than the younger age group.

**Conclusion:**

Repeated community-based surveys documented the effect of IHCI interventions in improving control at the community level. These results support the idea that IHCI strategies can possibly improve BP control in India and similar settings in Low- and Middle-income countries.

## Graphical abstract

**Figure:**
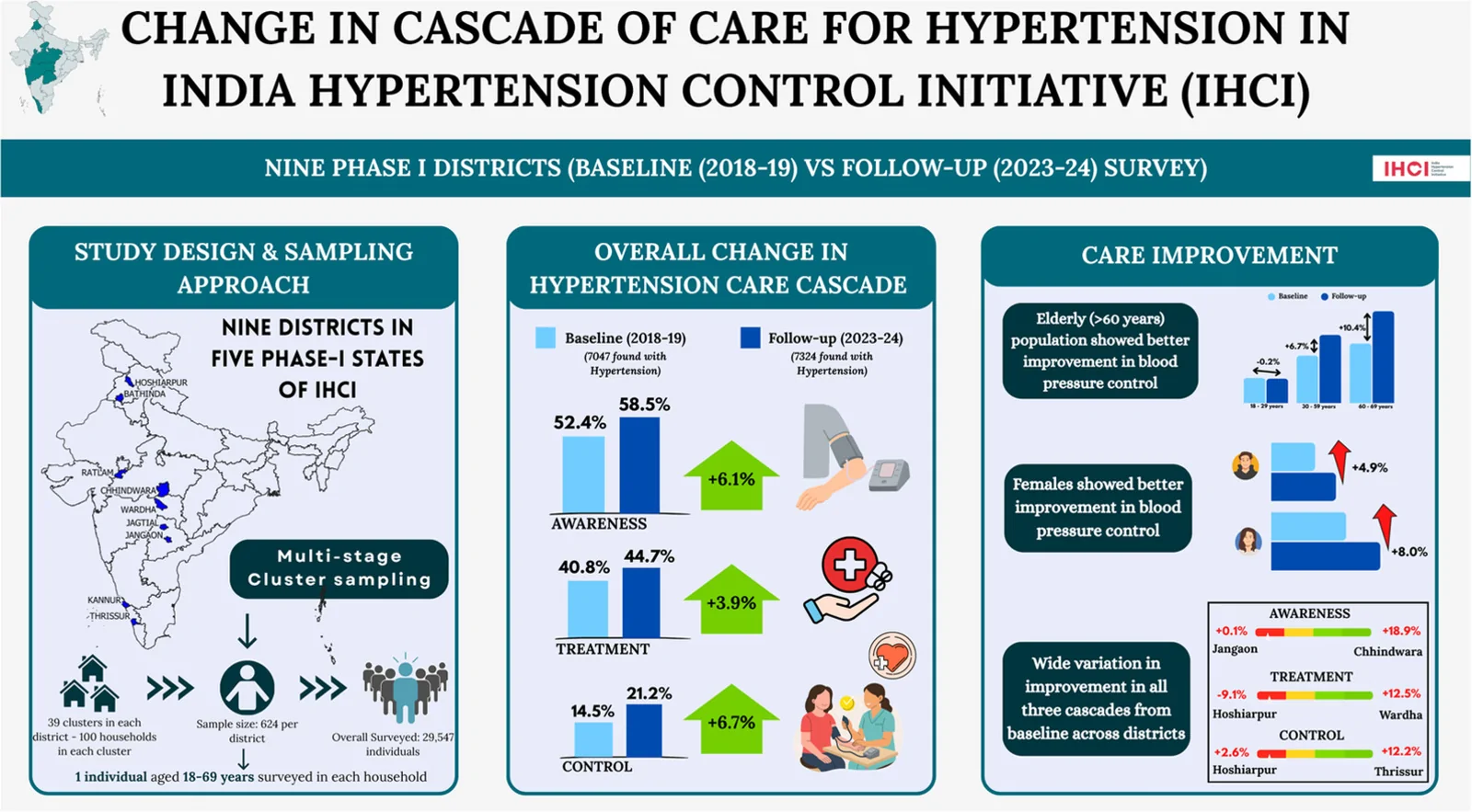

## 1 Introduction

The world's most populous countries are undergoing demographic and economic transitions, leading to rapid urbanization and the emergence of modern risks due to lifestyle changes. The strong behavioral risk factors due to lifestyle changes, in turn, increase the NCD burden [1]. Of the NCD burden, cardiovascular disease (CVD) is the leading cause of death, and high blood pressure is the major causative factor [2]. Globally, half of the population was aware of their hypertension status, nearly two-fifths were under treatment, and only one-fifth of them were under control [3]. Despite widespread hypertension screening campaigns and the wide availability of relatively inexpensive treatments, there are significant gaps in the level of cascade of care (awareness, treatment adherence, and control) of hypertension. Understanding this cascade at the population level is critical to plan more effective interventions [4].

India has an estimated over 200 million people with high blood pressure [5,6]. A national representative survey of India revealed that less than one-third (27.9%) were aware of their hypertension status, less than two-fifths (14.5%) were under treatment, and only 12.6% had blood pressure under control. The National Family Health Survey 5th round (2019–21) also documented less than one-third treatment coverage, with variations ranging from 13.6% (Madhya Pradesh) to 27.3% (Punjab) among all aware hypertensives, and control among individuals under treatment varied from 20.6% to 35.7% [7]. The National Program for Noncommunicable Diseases (NP-NCD) in India includes screening and treatment of hypertension at primary and secondary healthcare facilities [8]. Recognizing this rising burden and the need to improve the quality of the hypertension cascade of care, the India Hypertension Control Initiative (IHCI), a multi-partner initiative, was launched in 2017 based on the WHO HEARTS technical package [9]. The key intervention components of the program included: standardized treatment protocol, regulated medication supply of recommended protocol drugs, task sharing and team-based care, an information system for monitoring, and overall patient-centered care [10,11]. IHCI Phase I was implemented in 26 districts across five states (Telangana, Kerala, Madhya Pradesh, Maharashtra, and Punjab), where we observed a threefold increase in blood pressure (BP) control among hypertension patients under care at their public health facilities according to the facility-based data [12]. However, facility-based data do not provide a complete picture of hypertension control at the community level. Hence, in 2018–19, we conducted a baseline community survey in phase I districts to understand the population-level cascade of care and to document any changes in the pattern over the years due to the implementation of IHCI. The results of the baseline survey have been published elsewhere [13,7]. We observed substantial variation in baseline treatment and control across these five states, and this was also documented in the NFHS survey, where treatment coverage ranged from 15.3% to 21.2% across the IHCI survey states.

A repeat community-based survey assessed population-level awareness, treatment, and control of hypertension in the same nine phase I districts in 2023–24. The primary objective was to estimate the change in control at the community level among individuals with hypertension between baseline (2018–19) and follow-up (2023–24) surveys in nine phase-I districts of IHCI. The secondary objective was to assess changes in awareness and treatment among those with hypertension; the proportion of individuals under treatment and control among those aware of their hypertension status; and the change in mean systolic and diastolic blood pressure between the two surveys.

## 2 Methods

We conducted a repeat cross-sectional follow-up survey between December 2023 and November 2024 in the same nine (of the baseline survey) of the 25 Phase-I districts of the program across five states. Of these nine districts, two districts in each of the four states, Punjab (PB), Madhya Pradesh (MP), Telangana (TL), and Kerala (KL), and one district in Maharashtra (MH) were included [13]. The baseline survey, conducted prior to the implementation of IHCI, was completed in 2018–19, and our team subsequently published the findings.

### 2.1 Study population, sampling, and sample size

We included adults aged 18 to 69 years with raised blood pressure during the survey or who were already diagnosed with hypertension and currently under treatment. Eligible households were structures with a shared kitchen in which family members, including at least one individual aged 18–69 years, have been residing for more than six months. The individuals were included in the study regardless of whether they utilized public or private-sector health services.

We excluded households in which the team was unable to enumerate the members due to unavailability, refusal, lack of competent or appropriate respondents to provide information, or the absence of any eligible members in the household. We excluded the individuals who were not available for the interview after three attempts on different occasions, refused to participate, were reclassified as ineligible (based on age, period of stay, pregnancy status, mental disability, hearing loss, or terminally ill), or were unable to provide consent at the time of the survey.

We adopted a four-stage sampling strategy in our survey. Initially, survey clusters were selected using probability-proportional-to-size (household as the unit of size) systematic sampling [14]. We used our primary sampling units as census enumeration blocks (CEBs), which are the smallest operational geographical units for enumeration and typically cover about 100–125 households, as per the census of India frame used in India-based methodological studies. Thus, within each selected cluster, a CEB was chosen by probability proportional to size, and within each selected CEB, households were selected systematically. In each selected household, one individual was selected among eligible adults by simple random sampling.

The primary outcome of interest was to assess the change in blood pressure under control among the eligible adults from baseline to the follow-up survey. Secondary outcomes were a) the proportions of individuals aware of their hypertension status and the proportion under treatment among those with hypertension; b) the proportion treated and the proportion controlled among individuals aware of their hypertension status, and c) the mean change in systolic and diastolic blood pressure between surveys [15,16]. Assuming an increase in blood pressure control from 20% at baseline to 30% at follow-up among individuals with hypertension, with an intra-cluster correlation coefficient of 0.04 and a design effect of 1.6, the required sample size was estimated to be 624 adults with hypertension, with a 95% confidence interval and 90% power [16]. Considering a hypertension prevalence of 25% among adults aged 18–69 years, based on national estimates, the required total sample size was 2496 individuals [13]. We further adjusted this sample to account for non-response rates (20% at the household level and 20% at the individual level), resulting in a final sample size of 3900 adults per district. Details of sample size and sampling strategy have been published elsewhere.

### 2.2 Data collection

We used the Open Data Kit (ODK) Android-based tool for digital data collection [17]. In each selected cluster, enumeration was done, and a unique ID was provided to each household. In selected households, the age and gender of the eligible adults were recorded. From the listed eligible adults, the ODK-based program randomly selected one adult to participate in the survey. After obtaining informed consent, the trained field investigators measured the blood pressure of the selected individuals twice at 5-minute intervals using a digital, validated professional BP monitor (Omron HBP 1320). The team collected details of previous diagnoses and treatments for hypertension from each surveyed individual. A detailed interview was conducted with individuals with probable hypertension who had at least one of the two blood pressure measurements [systolic blood pressure (SBP) ≥ 140 mmHg or diastolic blood pressure (DBP) ≥ 90 mmHg], and those who reported a history of hypertension. Along with the detailed interview, their third BP reading, height, and weight were measured using a stadiometer (SECA 213) and a weighing scale (SECA 803), respectively.

### 2.3 Operational definitions

[18] Among those selected based on initially elevated blood pressure or prior diagnosis, hypertension was defined as the average of the second and third blood pressure readings, and the criteria included SBP ≥ 140 mmHg, DBP ≥ 90 mmHg, or treatment with antihypertensive medications in the previous two weeks. Awareness of hypertension was defined as an individual who has ever been told by any healthcare provider that they have high blood pressure, and those who have the average of second and third blood pressure readings of SBP ≥ 140 mmHg, DBP ≥ 90 mmHg. The treatment coverage included individuals with hypertension who had taken medications for the past two weeks. BP Control was defined as SBP ≤ 140 and DBP ≤ 90 mmHg.

### 2.4 Statistical analysis

The ODK-collected data was exported in comma-separated values (CSV) format and imported into Microsoft Excel. Key measurable outcomes, including hypertension prevalence, awareness, treatment coverage, and control, were analysed by district, age, gender, education, and occupation, expressed as proportions with 95% confidence intervals. After adjusting for household non-response at the cluster level and individual non-response at the district level, the appropriate sampling weights were calculated using a multi-stage sampling design. We projected the population aged 18–69 years for 2023 using 2011 and 2001 censuses, using the average exponential growth rate. Using the 2023 projected population, the calibrated sampling weight was calculated for each district. Complex sample weighted analysis was used to generate weighted frequencies and percentages, along with 95% confidence intervals, for each district's hypertension, awareness, treatment, and control outcome variables. Among individuals who are aware of their hypertension status, cluster-adjusted analysis was used to estimate treatment and control estimates [13]. We documented the change in the cascade of care for hypertension as proportions by district, observed in the baseline and follow-up surveys. To estimate the change in the proportion of blood pressure control between baseline and follow-up, we used predictive margins from a survey-weighted logistic regression model (svy: logit in Stata) to obtain marginal proportions, accounting for the cluster sampling design. We report both unadjusted estimates and adjusted estimated (age, sex, district, and comorbidities including diabetes status, cholesterol status, history of heart attack/stroke). Results for both estimates are expressed as percentages with 95% confidence intervals.

To assess changes in mean systolic and diastolic blood pressure between the two surveys, survey-based linear regression (svy: regress in Stata) was used, accounting for the cluster design. Analyses were conducted separately for four populations: all individuals interviewed, individuals with hypertension, individuals aware of their hypertension status, and individuals under antihypertensive treatment. The outcome was the mean difference in BP (Baseline minus follow-up), expressed in mmHg. Crude (unadjusted) and adjusted models have been reported, where the adjusted model has been controlled for age group, gender, district, and comorbidities, including diabetes, chronic kidney disease, and history of cardiovascular diseases. Results are presented as mean differences with 95% confidence intervals and t-statistics. A two-sided *p*-value of <0.05 was considered statistically significant.

Mean and standard deviation (SD) were calculated for age, systolic blood pressure, and diastolic blood pressure. We analysed the data using the software STATA SE (version 17.0) (StataCorp LLC, Texas, USA).

### 2.5 Human participants protection

The study was approved by the Institutional Human Ethics Committee of ICMR National Institute of Epidemiology, Chennai (NIE/IHEC/201709-02). We obtained written informed consent from the respondents before data collection. We used unique identifiers for the data collection and analysis. Identification details were not collected. The confidentiality of all participants was maintained.

## 3 Results

We surveyed 35,100 adults aged 18–69 years across nine districts during the follow-up survey. Of those surveyed, 32,063 (91.4%) responded at the household level, and among them, 29,547 completed their individual-level interview. Descriptions of the individuals interviewed in the follow-up survey are provided in Supplementary Tables 1 and 2.

Among those interviewed, 7324 (24.8%) had hypertension (Table 1). Of those with hypertension, 3116 (42.6%) were aged 45–59 years, 4186 (57.2%) were female, 1955 (26.7%) had no formal schooling, and 2746 (37.5%) were either homemakers or students (Table 2). The mean (SD) age of individuals with hypertension was 52 (11.2) years.

**Table 1:** Prevalence of hypertension by age, gender and nine phase-I districts (weighted estimated) in follow-up (2023–24) survey of India hypertension control initiative.

Prevalence			
	N (Total individuals responded)	n (Number of individuals identified with hypertension)	% (CI 95%)
Overall	29,547	7324	24.8% (24.0–25.6)
**Age group (in years)**			
18–44	16,175	1751	10.8% (10.2–11.5)
45–59	8744	3116	35.6% (34.3–37.0)
60–69	4628	2457	53.1% (51.4–54.8)

**Gender**			
Male	11,454	3138	27.4% (26.2–28.6)
Female	18,093	4186	23.1% (22.3–24.0)

**Prevalence (Weighted)**			
**Nine-Phase I Districts**	N (in millions)	n (95% CI) in 1000	% (CI 95%)

Hoshiarpur (Punjab)	1.11	310.2 (272.6–347.8)	27.9% (24.9–31.1)
Bathinda (Punjab)	1.12	284.3 (237.2–331.4)	25.5% (23.0–28.1)
Ratlam (MP)	1.05	241.0 (189.6–292.5)	22.9% (20.6–25.3)
Chhindwara (MP)	1.48	365.2 (313.7–416.6)	24.7% (22.6–27.0)
Wardha (MH)	0.92	177.8 (151.6–204.1)	19.4% (16.9–22.1)
Jagtial (Telangana)	0.72	151.3 (129.5–173.1)	21.1% (19.0–23.4)
Jangaon (Telangana)	0.38	97.5 (84.2–110.8)	25.6% (22.5–28.8)
Kannur (Kerala)	1.77	417.3 (377.4–457.2)	23.5% (21.9–25.3)
Thrissur (Kerala)	2.23	602.8 (532.6–673.0)	27.0% (25.3–28.8)

**Table 2:** Characteristics of individuals with hypertension by age, gender, education, and occupation participated in follow-up (2023–24) surveys in nine phase-I districts of India hypertension control initiative, India.

Characteristics		2023–24 (N = 7324)
	N (Total individuals responded)	%
**Age group (in years)**		
18–44	1751	23.9
45–59	3116	42.6
60–69	2457	33.6

**Gender**		
Male	3138	42.9
Female	4186	57.2

**Education Status**		
No Formal Schooling	1955	26.7
Upto Middle School (Upto 8)	2285	31.2
High School (9–10)	1494	20.4
Secondary School and Above	1579	21.6
No Response	11	0.2

**Occupation**		
Highly Skilled*	1189	16.2
Agricultural land owner	681	9.3
Manual Labour	2074	28.3
Homemaker/Student	2746	37.5
Others (Retired/Unemployed)	634	8.7

**Phase-I Districts**		
Hoshiarpur (Punjab)	902	12.3
Bathinda (Punjab)	770	10.5
Ratlam (MP)	727	9.9
Chhindwara (MP)	896	12.2
Wardha (MH)	673	9.2
Jagtial (Telangana)	729	10.0
Jangaon (Telangana)	910	12.4
Kannur (Kerala)	803	11.0
Thrissur (Kerala)	914	12.5

⁎ Professional/Business/Sales/Marketing/Self-employed.

Of the 7324 individuals with hypertension, 4284 (58.5%) were aware of their hypertension status, 3276 (44.7%) were receiving treatment, and 1551 (21.2%) were under BP control. The mean (SD) age of individuals with blood pressure under control was 57 (8.5) years. Control of blood pressure varied across the nine districts, from 34.1% in Thrissur (KL) to 7.8% in Ratlam (MP) (Supplementary Table 3).

During 2018–19, 7047 individuals with hypertension were included in the baseline survey across the same nine districts. The following sections describe changes in awareness, treatment, and control of hypertension between the baseline and follow-up surveys.

### 3.1 Change in awareness, treatment coverage, and control among hypertensive individuals

Overall, from 2018–19 to 2023–24, there was a 6.1% absolute increase in awareness of hypertension. This change ranged from 18.9% in Chhindwara (MP) to 0.1% in Jangaon (TL) (Supplementary Table 4).

Treatment coverage among individuals with hypertension improved by 3.9% in the follow-up survey (44.7%) compared to the baseline survey (40.8%). Improvement in treatment coverage was reported in seven of nine districts. Hoshiarpur (PB) and Kannur (KL) reported a decrease in treatment coverage in the follow-up period compared to the baseline. Among the seven districts that reported improvement, the increase in treatment coverage ranged from 12.5% in Wardha (MH) to 4.7% in Thrissur (KL) district (Supplementary Table 3).

We observed an overall 6.7% improvement in blood pressure control among individuals with hypertension. All nine districts had improvement in the control, with increases ranging from 2.6% in Hoshiarpur to 12.2% in Thrissur (Fig. 1). When adjusted for age, gender, district and other comorbidities (including diabetes, chronic kidney disease and history of cardiovascular diseases), the proportion of blood pressure control among individuals with hypertension increased from 14.7% (95% CI: 13.5% – 15.9%) to 20.9% (95% CI: 19.5% - 22.4%), with an absolute increase of 6.3% (95% CI: 4.8 – 7.7) from baseline and follow-up surveys (Supplementary Table 5).

**Fig. 1:**
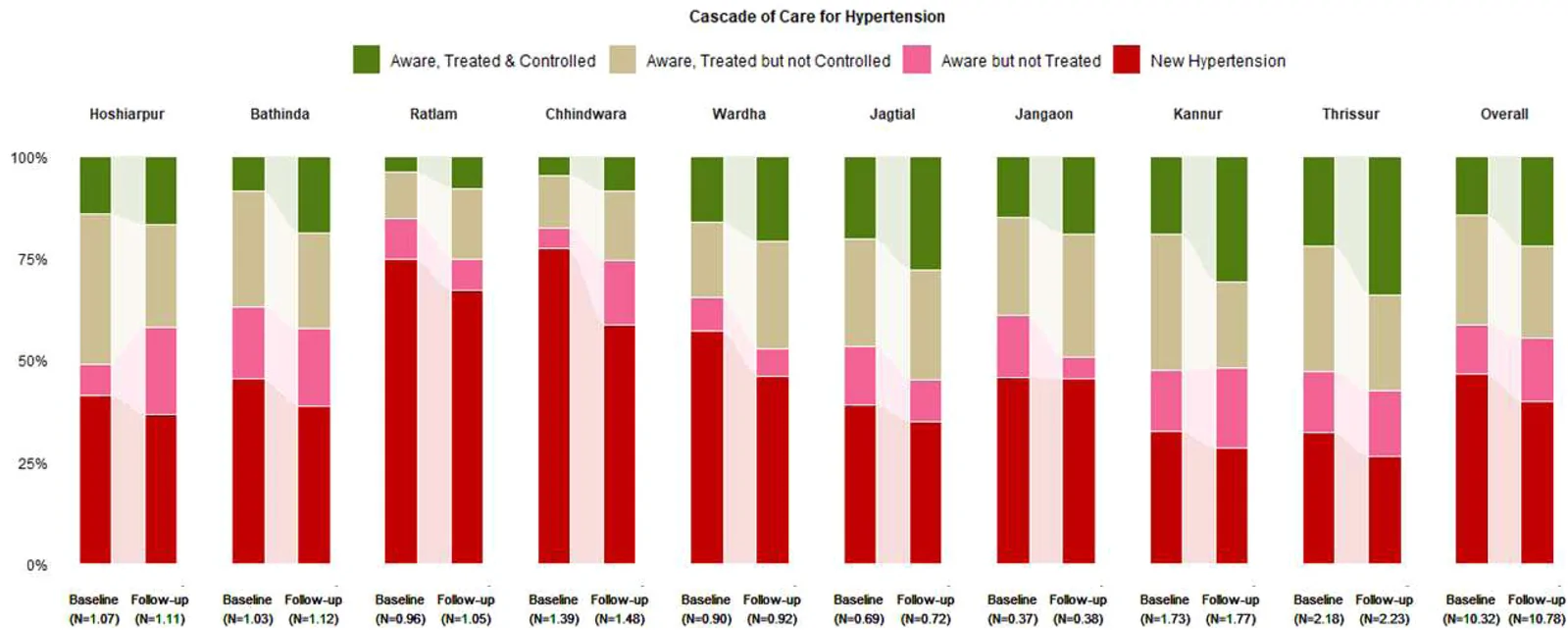
Awareness, treatment, and control of individuals with hypertension (weighted estimated) in baseline (2018–19) and follow-up (2023–24) survey in nine phase-I districts of India hypertension control initiative ^12.^ Note: Baseline data has already been published and is mentioned here for reference.

After adjusting for cluster design, we observed a significant decline in mean systolic BP among all individuals with hypertension (−4.7 mmHg; 95% CI: −5.5 to −3.9; *p* < 0.001) from baseline to follow-up (Supplementary Table 6). Across all districts, the 60–69 age group showed the highest increases in awareness (8%), treatment (7.5%), and control (10.4%) compared to other age groups. The improvement in awareness (from 59.3% to 66.1%), treatment (from 47.6% to 51.9%), and control (from 17.6% to 25.6%) was higher among females from baseline to follow-up (Fig. 2).

**Fig. 2:**
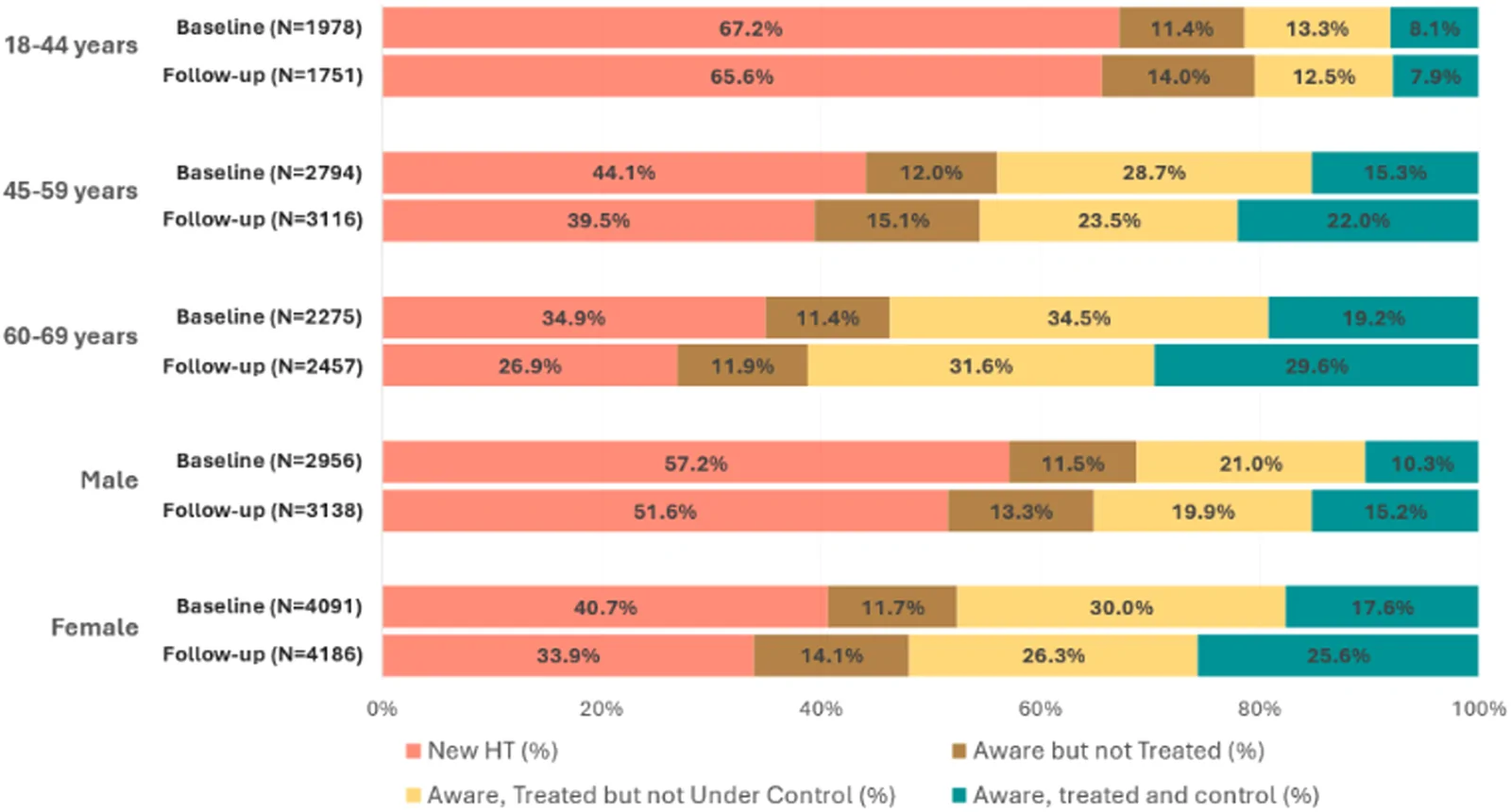
Awareness, Treatment, and Control by age, gender, education, and occupation among Individuals with Hypertension participated in Baseline (2018–19) and Follow-up (2023–24) in Nine Phase-I Districts of India Hypertension Control Initiative ^12.^ Note: Baseline Data has already been published and is mentioned here for reference.

### 3.2 Treatment coverage and control among individuals aware of their hypertension status

Among individuals aware of their hypertension status, six of nine districts showed improvement in treatment coverage (Table 3). The increase ranged from 19.5% in Ratlam to 0.3% in Thrissur. Among those aware, seven out of nine districts showed improvement in control, which ranged from 17.9% in Bathinda to 3.3% in Hoshiarpur.

**Table 3:** Treatment and control of hypertension among aware individuals by district (9 districts) comparison between baseline (2018–19) and follow-up survey (2023–24) in nine phase-i districts of India hypertension control initiative (12).

			Treatment			Control		
**State**	**District**	**Survey**	n	%	CI	n	%	CI
Punjab	Hoshiarpur	Baseline* (*N* = 604)	525	86.9	(82.3–90.5)	148	24.5	(20.4–29.2)
		Follow-up (*N* = 572)	377	65.9	(60.2–71.1)	159	27.8	(23.7–32.3)
		Difference		−21.0	(−27.9, −14.1)		3.3	(−2.8, 9.4)
	Bathinda	Baseline* (*N* = 472)	322	68.2	(61.7–74.1)	67	14.2	(10.4–19.1)
		Follow-up (*N* = 452)	316	69.9	(61.8–77.0)	145	32.1	(27.3–37.3)
		Difference		1.7	(−8.1, 11.5)		17.9	(11.3, 24.5)

Madhya Pradesh	Ratlam	Baseline* (*N* = 191)	115	60.2	(52.0–67.9)	30	15.7	(10.1–23.6)
		Follow-up (*N* = 242)	193	79.8	(70.3–86.7)	66	27.3	(21.1–34.5)
		Difference		19.5	(8.1, 30.9)		11.6	(1.5, 21.6)
	Chhindwara	Baseline* (*N* = 163)	135	82.8	(73.5–89.4)	37	22.7	(17.4–29.0)
		Follow-up (*N* = 384)	239	62.2	(53.6–70.1)	86	22.4	(17.9–27.7)
		Difference		−20.6	(−32.1, −9.2)		−0.3	(−8.4, 7.8)

Maharashtra	Wardha	Baseline* (*N* = 271)	223	82.3	(77.4–86.3)	109	40.2	(33.2–47.7)
		Follow-up (*N* = 366)	316	86.3	(80.9–90.4)	140	38.3	(33.4–43.3)
		Difference		4.1	(−2.4, 10.6)		−2.0	(−11.0, 7.1)

Telangana	Jagtial	Baseline* (*N* = 417)	311	74.6	(69.7–78.9)	145	34.8	(29.8–40.1)
		Follow-up (*N* = 478)	401	83.9	(78.8–87.9)	198	41.4	(36.7–46.3)
		Difference		9.3	(2.9, 15.7)		6.7	(−0.2, 13.5)
	Jangaon	Baseline* (*N* = 443)	343	77.4	(72.4–81.7)	130	29.4	(25.5–33.6)
		Follow-up (*N* = 531)	477	89.8	(84.7–93.3)	183	34.5	(30.0–39.2)
		Difference		12.4	(6.1, 18.7)		5.1	(−1.1, 11.3)

Kerala	Kannur	Baseline* (*N* = 531)	428	80.6	(76.5–84.1)	164	30.9	(26.5–35.6)
		Follow-up (*N* = 579)	420	72.5	(67.2–77.3)	251	43.4	(37.7–49.2)
		Difference		−8.1	(−14.4, −1.8)		12.5	(5.2, 19.7)
	Thrissur	Baseline* (*N* = 599)	471	78.6	(74.5–82.2)	193	32.2	(28.4–36.3)
		Follow-up (*N* = 680)	537	79.0	(75.1–82.4)	323	47.5	(41.8–53.3)
		Difference		0.3	(−5.0, 5.6)		15.3	(8.4, 22.1)

⁎ Baseline Data already published and mentioned here for reference.

Also, between baseline and follow-up surveys, the overall mean SBP (SD) among all aware hypertensives was reduced by 5.5 mmHg (150.6 (±20.9) to 145.1 (±20.8) mmHg), and the mean DBP (SD) changed by 0.2 mmHg (87.7 (±12.1) to 87.9 (±12.2) mmHg). The adjusted decline in mean SBP was 5.8 mmHg among individuals aware of their hypertension status (Supplementary Table 6). Among those aware and receiving antihypertensive treatment, the decline was even higher at 6.5 mmHg (95% CI: −7.7 to −5.2, *p* < 0.001). However, the changes in diastolic BP were not statistically significant across subgroups (Supplementary Table 6).

## 4 Discussion

Repeated surveys of the same communities in nine districts across five Indian states documented changes in the care cascade for hypertension during the IHCI implementation period. Objective data from both surveys on IHCI outcomes at the community level provide a more accurate measure of the program's population health benefits than facility-based surveys. National surveys indicate that the overall hypertension control in India is approximately 12.6% [6]. There are an estimated 200 million people with hypertension in India, and with the above-quoted control proportion, there are 25 million under control [5]. Overall control in IHCI districts was 21.2%. Assuming IHCI strategies are scaled countrywide on 200 million people with hypertension, it can lead to an increase in the number of people with controlled hypertension from 25 million to 42 million (a 17 million increase), preventing thousands of heart attacks, strokes, and deaths every year [19]. The evidence indicates that BP reduction among individuals with baseline SBP above 140 mmHg is associated with a significant reduction in CVD events and mortality.

Our study offers unique insights, as there have been limited data from India reporting community-level changes in the hypertension cascade of care across a larger geographical area (district-level) and collected at two time points before and after an intervention. Large variations in outcomes across districts emphasize the importance of district-level data for designing programmatic interventions. The wide variations recorded in our study across our five survey states were also consistent with recent national survey findings [7]. Over five years, overall, though there was an increase in awareness about their hypertension status, more than half of the individuals with hypertension were not under treatment, and a modest increase in community control of blood pressure was observed. Though community-based hypertension screening has increased over time, it has been reported to add much less to the effectiveness of primary healthcare services, and the transition of treatment to control requires ongoing care [20]. Districts with high awareness at baseline, such as Kannur, Thrissur, and Jangaon, demonstrated minimal improvement in awareness across two surveys despite ongoing community health worker-based screening under the NP-NCD program during this period. This suggests that traditional screening approaches may reach a plateau and not yield further improvement. In contrast, Chhindwara (MP) and Wardha (MH), which had low baseline awareness, showed remarkable improvement, possibly due to enhanced screening efforts by community-level health workers. A lack of adequate improvement in awareness could also be due to missed opportunities in primary care outpatient settings [21]. Low awareness and minimal improvement among males and the 18–44 years age group suggest the need for novel innovative strategies to reach these groups, possibly through workplaces or other suitable community-based settings.

The treatment coverage we recorded was effective, measuring whether individuals with hypertension were under treatment in the 2 weeks prior to the survey. The districts with a low baseline, such as Ratlam (MP) and Chhindwara (MP), recorded the largest increase in treatment coverage, indicating enhanced community utilization of services following the interventions. The districts in Kerala and Telangana with high baseline treatment coverage recorded small improvements at follow-up. This suggests that districts with a treatment proportion above 50% may require highly targeted strategies to reach groups with low treatment coverage, either by improving screening or by enhancing initiation/ retention in treatment among those already aware of the condition. Otherwise, treatment coverage also plateaus on continuing existing strategies over time. Districts Hoshiarpur (PB), Kannur (KL), and Chhindwara (MP) showed a decline in treatment coverage among those aware, indicating challenges with retention in care even after diagnosis and treatment initiation. Although we recorded improvements in treatment coverage across most districts, substantial variation persisted, possibly due to differences in health system capacity and health-seeking behavior. The variation in treatment coverage we recorded across states (ranging between 17.1% in MP to 54.7% in KL) was similar to the state-level estimates from the national survey. The treatment coverage documented in NFHS 5th round (2019–21) ranged between 8.8% in MP to 23.6% in Kerala. Improving treatment coverage in India and other low- and middle-income countries is challenging due to a fragmented healthcare delivery system, inadequate access to cost-effective care, and limited patient-centered care. The 'patients' experience, including lack of general awareness, lifestyle habits, and attitude towards medication adherence, was another challenge [22]. Inadequate drug availability and challenges in accessing healthcare facilities in some parts of states like Madhya Pradesh and Telangana also contributed to poor treatment coverage [23,24]. Additional workload, poor efforts in counselling care, and socio-economic barriers, including out-of-pocket expenditure, patient-provider communication gaps, and longer waiting time at clinics, have affected consistent care, undermining treatment coverage at the population level in states like Kerala and Maharashtra.

Overall, we observed improvements in BP control across all study districts. Various studies have documented changes in BP control over time. A study in Haryana state, northern India, reported a 4.6% increase over 8 years [25,26]. Jaipur Heart Watch Study documented a 19% change in BP control over 25 years. The change in control in both studies translates to less than 1% per year. National surveys documented wider variations in BP control among those treated across IHCI survey districts, ranging from 4.2% in Ratlam to 30.3% in Thrissur [7]. We documented a 6.7% change over approximately 5 years, including the COVID-19 pandemic. The improvement observed during this period was possibly catalyzed by the IHCI interventions, which accelerated the progress under the NP-NCD program. It was interesting to note that control improved across districts, regardless of whether treatment coverage increased. In Thrissur (KL) and Bathinda (PB), there was a remarkable increase in BP control with higher treatment coverage. On the contrary, despite the decline in treatment coverage, BP control improved among those treated in Kannur (KL) and Hoshiarpur (PB), possibly due to higher-quality care. Though Wardha (MH) recorded an improvement in awareness and treatment coverage, BP control among those treated declined, possibly due to drug shortages [27]. The extent to which interventions are implemented influences outcomes at different levels of the cascade.

Variations in the care cascade across age groups and genders reveal the characteristics of beneficiaries who sought care at public-sector facilities. Compared with younger age groups, the hypertension care cascade was more effectively improved in the elderly population. This improvement is similar to cascade studies conducted in the past two decades [[21], [22], [23], [24], [25], [26], [27], [28], [29]]. The younger age group showed no improvement in the cascade [30]. The females showed greater improvement at all levels of the cascade than the males, consistent with the literature from the southern state of Kerala, where a similar pattern was documented, possibly due to greater utilization of the healthcare system.

### 4.1 Limitations

Our study has a few limitations. We did not survey the non-intervention districts due to resource constraints. We recorded individuals who were aware, under treatment, and in the control group at the time of the survey. Cross-sectional surveys did not capture the dynamic changes in care that occurred over the project period, spanning 2020 to 2022, including the COVID-19 pandemic. However, the cross-sectional survey of the cascade is suitable for surveillance trend analysis and assessing public health program performance in a limited-resource setting. We did not resurvey the same households or individuals; however, we conducted the resurvey in the same clusters to ensure comparability of the findings.

Differences in the improvement and coverage of awareness, treatment, and BP control across the districts were probably due to varied levels of implementation, health system factors, socio-cultural factors such as health-seeking behavior, and program management [31]. We could not document the reasons behind these differences, which require a more in-depth qualitative inquiry. One of the challenges encountered at the field level was obtaining an accurate history of diagnoses and treatments. However, dedicated and trained survey investigators, with supportive supervision, ensured that the best possible data were collected. We employed a strict definition of treatment over the previous two weeks to minimize misclassification. We measured BP twice for all, and measured the third only among those for whom one of the first two was above normal or who were on treatment for hypertension [32]. This approach was consistent with the evidence from a large national survey in India, which documented that one reading was reasonable for people with normal BP.

## 5 Conclusion and recommendation

Overall, our study concluded that in IHCI-implemented districts, there were improvements in the hypertension awareness, treatment, and control, hence providing evidence for scaling these interventions. Variations in improvement across districts reflected diverse health systems and patient behaviors, necessitating locally relevant adaptations in the interventions to address the cascade at various stages. We recommend a population-level cascade-of-care monitoring as a standard method for tracking the impact of hypertension interventions. As the survey is resource-intensive and requires a higher level of expertise, it is difficult to scale in large, resource-constrained countries. It might be feasible to use the surveys to track progress in the sentinel districts at 3–5-year intervals. Additionally, it is crucial to enhance facility-level information systems to enable the public and private sectors to obtain real-time estimates of treatment and control. A combination of facility and population surveillance is required for optimal resource allocation and understanding the impact. We also recommend in-depth qualitative research to explore the health system and patient-level factors that influence screening, health-seeking for treatment, and long-term retention in care.

## Sources of funding

WHO and the Indian Council of Medical Research (ICMR), India, together funded the IHCI project

## CRediT authorship contribution statement

**Parasuraman Ganeshkumar:** Writing – review & editing, Writing – original draft, Supervision, Formal analysis, Data curation. **Maanasa Rajagopalan:** Writing – review & editing, Writing – original draft, Formal analysis, Data curation. **Mosoniro Kriina:** Writing – review & editing, Writing – original draft, Formal analysis, Data curation. **Jayashree Somasundaram:** Writing – review & editing, Writing – original draft, Formal analysis, Data curation. **Jayashree Kalva:** Writing – review & editing, Writing – original draft, Formal analysis, Data curation. **Navaneeth S Krishna:** Writing – review & editing, Supervision, Project administration. **Kannamkottapilly Chandrasekharan Prajitha:** Writing – review & editing, Supervision, Project administration. **Sonie Sait:** Writing – review & editing, Supervision, Project administration. **Vettrichelvan Venkatasamy:** Writing – review & editing, Supervision, Software, Project administration. **Santhiya Venkatesan:** Writing – review & editing, Supervision, Project administration. **Sugavathy A:** Writing – review & editing, Supervision, Project administration. **Sailaja Bitragunta:** Writing – review & editing, Supervision, Project administration. **Meenakshi Sharma:** Writing – review & editing, Methodology, Conceptualization. **Roopa Shivashankar:** Writing – review & editing, Supervision, Project administration. **Pragati Pragya:** Writing – review & editing, Supervision, Project administration. **Sampada D Bangar:** Writing – review & editing, Methodology, Conceptualization. **Tapas Chakma:** Writing – review & editing, Supervision, Project administration. **Abhishek Kunwar:** Supervision, Project administration. **Amol Wankhede:** Writing – review & editing, Supervision, Project administration. **Rupali Bharadwaj:** Writing – review & editing, Supervision, Project administration. **Vishwajit Bharadwaj:** Writing – review & editing, Supervision, Project administration. **Sravan Kumar Reddy T:** Writing – review & editing, Supervision, Project administration. **Navneet Kishore:** Writing – review & editing, Supervision, Project administration. **Bidisha Das:** Writing – review & editing, Supervision, Project administration. **Yannick Puthussery:** Writing – review & editing, Supervision, Project administration. **Deenadayalan Chandran:** Writing – review & editing, Supervision, Project administration. **Branch Immanuel A:** Writing – review & editing, Supervision, Project administration. **Sandeep S Gill:** Writing – review & editing, Validation, Supervision, Project administration, Investigation. **Ashu Gupta:** Writing – review & editing, Validation, Supervision, Project administration, Investigation. **Bipin Gopal:** Writing – review & editing, Validation, Supervision, Project administration, Investigation. **Ramakrishna Kodirekkala:** Writing – review & editing, Validation, Supervision, Project administration, Investigation. **Jagannath Reddy M:** Writing – review & editing, Validation, Supervision, Project administration, Investigation. **Sarita Hazare:** Writing – review & editing, Validation, Supervision, Project administration, Investigation. **Namita Neelkanth:** Writing – review & editing, Validation, Supervision, Project administration, Investigation. **Gagandeep Singh Grover:** Writing – review & editing, Validation, Supervision, Project administration, Investigation. **Anupam Khungar Pathni:** Writing – review & editing, Validation, Supervision, Project administration, Investigation. **Prabhdeep Kaur:** Writing – review & editing, Writing – original draft, Supervision, Methodology, Formal analysis, Conceptualization.

## Declaration of competing interest

The authors declare that they have no known competing financial interests or personal relationships that could have appeared to influence the work reported in this paper.
