# Supplementary material for: Change in cascade of care among individuals with hypertension – India hypertension control initiative, 2018-2024
Source: Am J Prev Cardiol. 2026 Jun 17;30:101705. doi: 10.1016/j.ajpc.2026.101705 (PMC13631880; doi:10.1016/j.ajpc.2026.101705)
Supplement: Supplementary file 1 [file mmc1.docx]

**SUPPLEMENTARY FILES**

**Supplementary Table 1: Characteristics of Individuals participated in Follow-up (2023-24) survey in Nine Phase-I Districts of India Hypertension Control Initiative**

|  | **2023-24 (N=29547)** | |
| --- | --- | --- |
|  | n | % |
| **Age group (in years)** |  |  |
| 18-44 | 16,175 | 54.7 |
| 45-59 | 8,744 | 29.6 |
| 60-69 | 4,628 | 15.7 |
| **Gender** |  |  |
| Male | 11,454 | 38.8 |
| Female | 18,093 | 61.2 |
| **District** |  |  |
| Hoshiarpur (Punjab) | 3,200 | 10.8 |
| Bathinda (Punjab) | 3,154 | 10.7 |
| Ratlam (MP) | 3,268 | 11.1 |
| Chhindwara (MP) | 3,526 | 11.9 |
| Wardha (MH) | 3,294 | 11.2 |
| Jagtial (Telangana) | 3,281 | 11.1 |
| Jangaon (Telangana) | 3,289 | 11.1 |
| Kannur (Kerala) | 3,239 | 11.0 |
| Thrissur (Kerala) | 3,296 | 11.2 |

**Supplementary Table 2: Characteristics of Individuals with Hypertension participated in the Follow-up (2023-24) survey by district in the Nine Phase-I districts of India Hypertension Control Initiative**

| **District** |  | **Age group (In years)** | | | | | | **Gender** | | | | **Education** | | | | | | | | | | | **Occupation** | | | | | | | | | | |
| --- | --- | --- | --- | --- | --- | --- | --- | --- | --- | --- | --- | --- | --- | --- | --- | --- | --- | --- | --- | --- | --- | --- | --- | --- | --- | --- | --- | --- | --- | --- | --- | --- | --- |
|  |  | **18-44** | | **45-59** | | **60-69** | | **Male** | | **Female** | | **No formal** | | **Upto Middle School** | | **High school** | | **Secondary and above** | | **No Response** | | | **Highly Skilled** | | **Agricultural land owner** | | | **Manual Labour** | | **Homemaker/Student** | | **Others (Retired/ Unemployed)** | |
|  | **N** | **n** | **%** | **n** | **%** | **n** | **%** | **n** | **%** | **n** | **%** | **n** | **%** | **n** | **%** | **n** | **%** | **n** | **%** | **n** | **%** | **n** | | **%** | **n** | **%** | **n** | | **%** | **n** | **%** | **n** | **%** |
| Hoshiarpur | 902 | 243 | 26.9% | 397 | 44.0% | 262 | 29.0% | 352 | 39.0% | 550 | 61.0% | 98 | 10.9% | 304 | 33.7% | 252 | 27.9% | 248 | 27.5% | 0 | 0.0% | 169 | | 18.7% | 35 | 3.9% | 134 | | 14.9% | 480 | 53.2% | 84 | 9.3% |
| Bathinda | 770 | 269 | 34.9% | 284 | 36.9% | 217 | 28.2% | 359 | 46.6% | 411 | 53.4% | 295 | 38.3% | 232 | 30.1% | 122 | 15.8% | 120 | 15.6% | 1 | 0.1% | 128 | | 16.6% | 78 | 10.1% | 122 | | 15.8% | 413 | 53.6% | 29 | 3.8% |
| Ratlam | 727 | 261 | 35.9% | 279 | 38.4% | 187 | 25.7% | 368 | 50.6% | 359 | 49.4% | 260 | 35.8% | 253 | 34.8% | 79 | 10.9% | 135 | 18.6% | 0 | 0.0% | 134 | | 18.4% | 120 | 16.5% | 205 | | 28.2% | 201 | 27.6% | 67 | 9.2% |
| Chhindwara | 896 | 294 | 32.8% | 391 | 43.6% | 211 | 23.5% | 441 | 49.2% | 455 | 50.8% | 283 | 31.6% | 347 | 38.7% | 105 | 11.7% | 153 | 17.1% | 8 | 0.9% | 109 | | 12.2% | 95 | 10.6% | 345 | | 38.5% | 313 | 34.9% | 34 | 3.8% |
| Wardha | 673 | 135 | 20.1% | 286 | 42.5% | 252 | 37.4% | 298 | 44.3% | 375 | 55.7% | 76 | 11.3% | 230 | 34.2% | 137 | 20.4% | 229 | 34.0% | 1 | 0.1% | 113 | | 16.8% | 114 | 16.9% | 209 | | 31.1% | 183 | 27.2% | 54 | 8.0% |
| Jagtial | 729 | 148 | 20.3% | 336 | 46.1% | 245 | 33.6% | 297 | 40.7% | 432 | 59.3% | 403 | 55.3% | 173 | 23.7% | 86 | 11.8% | 66 | 9.1% | 1 | 0.1% | 108 | | 14.8% | 109 | 15.0% | 294 | | 40.3% | 150 | 20.6% | 68 | 9.3% |
| Jangaon | 910 | 205 | 22.5% | 397 | 43.6% | 308 | 33.8% | 380 | 41.8% | 530 | 58.2% | 476 | 52.3% | 185 | 20.3% | 126 | 13.8% | 123 | 13.5% | 0 | 0.0% | 129 | | 14.2% | 117 | 12.9% | 338 | | 37.1% | 252 | 27.7% | 74 | 8.1% |
| Kannur | 803 | 87 | 10.8% | 347 | 43.2% | 369 | 46.0% | 270 | 33.6% | 533 | 66.4% | 35 | 4.4% | 292 | 36.4% | 264 | 32.9% | 212 | 26.4% | 0 | 0.0% | 116 | | 14.4% | 4 | 0.5% | 203 | | 25.3% | 385 | 47.9% | 95 | 11.8% |
| Thrissur | 914 | 109 | 11.9% | 399 | 43.7% | 406 | 44.4% | 373 | 40.8% | 541 | 59.2% | 29 | 3.2% | 269 | 29.4% | 323 | 35.3% | 293 | 32.1% | 0 | 0.0% | 183 | | 20.0% | 9 | 1.0% | 224 | | 24.5% | 369 | 40.4% | 129 | 14.1% |

**Supplementary Table 3: Awareness, Treatment, and Control of Hypertension (weighted estimated) by District (9 districts) comparison between Baseline (2018-19) and Follow-up Survey (2023-24) in Nine Phase-I districts of India Hypertension Control Initiative** ^12^

|  |  |  | **Awareness** | | | **Treatment** | | | | **Control** | | **Uncontrolled HT** | | |
| --- | --- | --- | --- | --- | --- | --- | --- | --- | --- | --- | --- | --- | --- | --- |
| **State** | **District** | **Survey**  **(in 1000)** | n (in 1000) | % | CI | n (in 1000) | % | CI | n (in 1000) | | % | CI | n (in 1000) | % |
| Punjab | Hoshiarpur | Baseline* (N=354.8) | 208.0 (178.6 - 237.4) | 58.6 | (54.3 - 62.8) | 180.8  (157.0 - 204.6) | 51.0 | (46.5 - 55.4) | 49.9  (37.4 - 62.4) | | 14.1 | (11.2 - 17.5) | 304.9 | 28.4 |
|  |  | Follow-up (N=310.2) | 196.2 (168.2 - 224.2) | 63.3 | (58.7 - 67.6) | 130.1 (106.8 - 153.4) | 41.9 | (37.6 - 46.4) | 51.8  (40.8 - 62.8) | | 16.7 | (14.2 - 19.5) | 258.4 | 23.2 |
|  |  | Difference |  | 4.7 |  |  | -9.1 |  |  | | 2.6 |  |  | -5.2 |
|  | Bathinda | Baseline* (N=295.6) | 161.6 (135.4 - 187.8) | 54.7 | (51.2 - 58.0) | 109.4  (86.2 - 132.6) | 37.0 | (32.1 - 42.3) | 25.1  (13.5 - 36.7) | | 8.5 | (5.8 - 12.3) | 270.5 | 26.2 |
|  |  | Follow-up (N=284.3) | 173.9 (132.2 - 215.6) | 61.2 | (54.6 - 67.3) | 120.2  (86.4 - 154.0) | 42.3 | (35.4 - 49.5) | 53.3  (38.0 - 68.5) | | 18.7 | (15.4 - 22.6) | 231.1 | 20.7 |
|  |  | Difference |  | 6.5 |  |  | 5.3 |  |  | | 10.2 |  |  | -5.5 |
| Madhya Pradesh | Ratlam | Baseline* (N=187.2) | 47.4 (35.4 - 59.5) | 25.3 | (20.4 - 31.0) | 28.7  (20.5 - 36.8) | 15.3 | (11.9 - 19.4) | 7.3  (3.1 - 11.5) | | 3.9 | (2.2 - 6.8) | 179.9 | 18.6 |
|  |  | Follow-up (N=241.0) | 79.6 (56.0 - 103.2) | 33.0 | (27.9 - 38.6) | 60.9  (43.2 - 78.7) | 25.3 | (20.4 - 31.0) | 18.9  (12.6 - 25.3) | | 7.8 | (5.6 - 10.9) | 222.1 | 21.1 |
|  |  | Difference |  | 7.7 |  |  | 10.0 |  |  | | 3.9 |  |  | 2.5 |
|  | Chhindwara | Baseline* (N=312.4) | 70.5 (44.9 - 96.2) | 22.6 | (17.1 - 29.3) | 54.6  (34.8 - 74.5) | 17.5 | (13.1 - 23.0) | 14.8  (7.9 - 21.7) | | 4.7 | (3.2 - 7.0) | 297.6 | 21.4 |
|  |  | Follow-up (N=365.2) | 151.5 (118.4 - 184.5) | 41.5 | (35.4 - 47.9) | 93.4  (68.6 - 118.1) | 25.6 | (20.5 - 31.4) | 31.0  (21.1 - 41.0) | | 8.5 | (6.3 - 11.3) | 334.1 | 22.6 |
|  |  | Difference |  | 18.9 |  |  | 8.1 |  |  | | 3.8 |  |  | 1.2 |
| Maharashtra | Wardha | Baseline* (N=160.4) | 68.5 (49.9 - 87.2) | 42.7 | (36.5 - 49.2) | 55.7 (40.7 - 70.8) | 34.7 | (29.4 - 40.6) | 26.1  (17.3 – 35.0) | | 16.3 | (12.5 - 21.0) | 134.3 | 15.0 |
|  |  | Follow-up (N=177.8) | 95.8 (76.5 - 115.0) | 53.9 | (48.7 - 58.9) | 83.9 (64.3 - 103.5) | 47.2 | (41.0 - 53.5) | 36.9  (25.8 - 47.9) | | 20.7 | (16.8 - 25.3) | 140.9 | 15.3 |
|  |  | Difference |  | 11.2 |  |  | 12.5 |  |  | | 4.4 |  |  | 0.3 |
| Telangana | Jagtial | Baseline* (N=128.9) | 78.5 (65.6 - 91.3) | 60.9 | (55.9 - 65.6) | 60.0 (48.7 - 71.4) | 46.6 | (41.7 - 51.5) | 26.0  (20.3 - 31.7) | | 20.2 | (16.9 - 24.0) | 102.9 | 15.0 |
|  |  | Follow-up (N=151.3) | 98.4 (84.0 - 112.7) | 65.0 | (58.9 - 70.7) | 83.1 (70.0 - 96.3) | 54.9 | (48.8 - 60.9) | 42.2  (34.6 - 49.8) | | 27.9 | (23.7 - 32.5) | 109.1 | 15.2 |
|  |  | Difference |  | 4.1 |  |  | 8.3 |  |  | | 7.7 |  |  | 0.2 |
|  | Jangaon | Baseline* (N=82.1) | 44.7 (37.2 - 52.1) | 54.4 | (49.4 - 59.3) | 32.0 (26.9 – 37.0) | 38.9 | (34.2 - 43.9) | 12.3  (9.6 – 15.0) | | 15.0 | (12.3 - 18.1) | 69.8 | 18.6 |
|  |  | Follow-up (N=97.5) | 53.1 (45.5 - 60.7) | 54.5 | (50.1 - 58.8) | 48.1 (41.4 - 54.8) | 49.4 | (44.7 - 54.1) | 18.5  (15.0 - 22.0) | | 19.0 | (15.5 - 23.0) | 79.0 | 20.7 |
|  |  | Difference |  | 0.1 |  |  | 10.5 |  |  | | 4.0 |  |  | 2.1 |
| Kerala | Kannur | Baseline* (N=384.9) | 259.3 (234.8 - 283.9) | 67.4 | (62.3 - 72.1) | 202.4 (180.7 – 224.0) | 52.6 | (47.1 - 58.0) | 73.7  (60.5 - 86.8) | | 19.1 | (15.6 - 23.2) | 311.2 | 18.0 |
|  |  | Follow-up (N=417.3) | 299.1 (268.8 - 329.3) | 71.7 | (66.4 - 76.4) | 217.1 (191.2 - 243.1) | 52.0 | (46.3 - 57.8) | 128.4 (107.8 - 148.9) | | 30.8 | (25.6 - 36.4) | 288.9 | 16.3 |
|  |  | Difference |  | 4.3 |  |  | -0.6 |  |  | | 11.7 |  |  | -1.7 |
|  | Thrissur | Baseline* (N=573.7) | 388.6 (351.6 - 425.6) | 67.7 | (63.9 - 71.4) | 303.4 (271.3 - 335.5) | 52.9 | (48.6 - 57.1) | 125.9 (104.6 - 147.2) | | 21.9 | (18.8 - 25.4) | 447.8 | 20.5 |
|  |  | Follow-up (N=602.8) | 444.2 (396.8 - 491.7) | 73.7 | (69.5 - 77.5) | 347.1 (310.1 - 384.1) | 57.6 | (52.6 - 62.4) | 205.4 (172.9 - 237.9) | | 34.1 | (29.2 - 39.3) | 397.4 | 17.8 |
|  |  | Difference |  | 6.0 |  |  | 4.7 |  |  | | 12.2 |  |  | -2.7 |

*Baseline Data already published and mentioned here for reference

**Supplementary Table 4: Awareness, Treatment and Control by age, gender, education and occupation among Individuals with Hypertension participated in Follow-up (2023-24) in Nine Phase-I Districts of India Hypertension Control Initiative**

|  | **Awareness** | | | **Treatment** | | | | **Control** | | |
| --- | --- | --- | --- | --- | --- | --- | --- | --- | --- | --- |
|  | N | n | % | N | n | % | N | | n | % |
| **Overall** | 7,324 | 4,284 | 58.5% | 7,324 | 3,276 | 44.7% | 7,324 | | 1,551 | 21.2% |
| **Age group (in years)** |  |  |  |  |  |  |  | |  |  |
| 18-44 | 1,751 | 602 | 34.4% | 1,751 | 357 | 20.4% | 1,751 | | 139 | 7.9% |
| 45-59 | 3,116 | 1,886 | 60.5% | 3,116 | 1,416 | 45.4% | 3,116 | | 685 | 22.0% |
| 60-69 | 2,457 | 1,796 | 73.1% | 2,457 | 1,503 | 61.2% | 2,457 | | 727 | 29.6% |
| **Gender** |  |  |  |  |  |  |  | |  |  |
| Male | 3,138 | 1,519 | 48.4% | 3,138 | 1,103 | 35.1% | 3,138 | | 478 | 15.2% |
| Female | 4,186 | 2,765 | 66.1% | 4,186 | 2,173 | 51.9% | 4,186 | | 1,073 | 25.6% |
| **Education Status** |  |  |  |  |  |  |  | |  |  |
| No formal schooling | 1,955 | 1,140 | 58.3% | 1,955 | 900 | 46.0% | 1,955 | | 387 | 19.8% |
| Up to Middle School (Up to Class VIII) | 2,285 | 1,339 | 58.6% | 2,285 | 1,005 | 44.0% | 2,285 | | 461 | 20.2% |
| High School (9-10) | 1,494 | 942 | 63.1% | 1,494 | 694 | 46.5% | 1,494 | | 359 | 24.0% |
| Secondary School and above | 1,579 | 856 | 54.2% | 1,579 | 674 | 42.7% | 1,579 | | 344 | 21.8% |
| No response | 11 | 7 | 63.6% | 11 | 3 | 27.3% | 11 | | 0 | 0.0% |
| **Occupation** |  |  |  |  |  |  |  | |  |  |
| Highly skilled* | 1,189 | 630 | 53.0% | 1,189 | 466 | 39.2% | 1,189 | | 217 | 18.3% |
| Agricultural land owner | 681 | 327 | 48.0% | 681 | 253 | 37.2% | 681 | | 111 | 16.3% |
| Manual Labour | 2,074 | 954 | 46.0% | 2,074 | 659 | 31.8% | 2,074 | | 276 | 13.3% |
| Homemaker/Student | 2,746 | 1,913 | 69.7% | 2,746 | 1,507 | 54.9% | 2,746 | | 746 | 27.2% |
| Others (Retired/Unemployed) | 634 | 460 | 72.6% | 634 | 391 | 61.7% | 634 | | 201 | 31.7% |

*****Professional/Business/Sales/Marketing/Self-employed

**Supplementary Table 5: Absolute difference in proportion of blood pressure control among individuals with Hypertension from baseline (N=7,047) to follow-up (N=7,324) in Nine Phase-I districts of India Hypertension Control Initiative**

|  | **Survey Round** | **Proportion** | **t** | **P > \|t\|** | **95% CI** |
| --- | --- | --- | --- | --- | --- |
| **Unadjusted** | Baseline | 14.5 | 24.0 | 0.000 | 13.3-15.7 |
|  | Follow-up | 21.2 | 27.4 | 0.000 | 19.7-22.7 |
|  | **Difference** | **6.7** |  |  | **5.1-8.2** |
| **Adjusted for age, gender** | Baseline | 14.7 | 24.2 | 0.000 | 13.5-15.9 |
|  | Follow-up | 20.9 | 27.5 | 0.000 | 19.5-22.4 |
|  | **Difference** | **6.3** |  |  | **4.8-7.7** |
| **Adjusted for age, gender, district, comorbidities*** | Baseline | 14.7 | 23.7 | 0.000 | 13.5-15.9 |
|  | Follow-up | 21.0 | 27.6 | 0.000 | 19.5-22.4 |
|  | **Difference** | **6.3** |  |  | **4.7-7.7** |

Notes: Estimates from cluster-adjusted logistic regression. All values expressed as percentages. *Comorbidities include diabetes, chronic kidney disease, history of cardiovascular disease

**Supplementary Table 6. Change in Mean Systolic and Diastolic Blood Pressure Between Baseline and Follow-up in Nine Phase-I Districts of India Hypertension Control Initiative**

| **Population** | **Model** | **Systolic BP (mmHg)** | | | | | | **Diastolic BP (mmHg)** | | | | | |
| --- | --- | --- | --- | --- | --- | --- | --- | --- | --- | --- | --- | --- | --- |
|  |  | **Baseline  mean** | **Follow-up  mean** | **Change †**  **(95% CI)** | **t** | **P > t** | **Baseline  mean** | | **Follow-up  mean** | **Change** **†**  **(95% CI)** | **t** | **P > t** |  |
| **All individuals with BP reading (N=15,936)** | **Crude** | 150.3 | 145.3 | **−5.0 (−5.9, −4.1)** | -10.49 | **<0.001** | 88.9 | | 89.2 | **0.3 (−0.2, 0.8)** | 1.15 | 0.253 |  |
|  | **Adjusted*** | 150.4 | 145.1 | **−5.3 (−6.2, −4.3)** | -10.92 | **<0.001** | 88.9 | | 89.3 | **0.4 (−0.1, 0.9)** | 1.65 | 0.099 |  |
| **Individuals with hypertension (N=14,371)** | **Crude** | 152.1 | 147.5 | **−4.6 (−5.4, −3.8)** | -11.27 | **<0.001** | 89.6 | | 90.3 | **0.7 (0.2, 1.1)** | 2.66 | **0.008** |  |
|  | **Adjusted*** | 152.1 | 147.4 | **−4.7 (−5.5, −3.9)** | -11.45 | **<0.001** | 89.5 | | 90.4 | **0.8 (0.4, 1.3)** | 3.73 | **<0.001** |  |
| **Individuals aware of their hypertension status (N=7,975)** | **Crude** | 150.4 | 144.7 | **−5.7 (−6.7, −4.6)** | -10.22 | **<0.001** | 87.3 | | 87.2 | **−0.1 (−0.7, 0.6)** | -0.20 | 0.844 |  |
|  | **Adjusted*** | 150.5 | 144.7 | **−5.8 (−6.9, −4.7)** | -10.51 | **<0.001** | 87.3 | | 87.3 | **−0.02 (−0.6, 0.6)** | -0.06 | 0.954 |  |
| **Individuals on antihypertensive treatment (N=6,149)** | **Crude** | 147.6 | 141.2 | **−6.4 (−7.6, −5.2)** | -10.33 | **<0.001** | 85.4 | | 84.9 | **−0.5 (−1.3, 0.2)** | -1.40 | 0.163 |  |
|  | **Adjusted*** | 147.7 | 141.2 | **−6.5 (−7.7, −5.2)** | -10.46 | **<0.001** | 85.4 | | 84.9 | **−0.5 (−1.2, 0.2)** | -1.39 | 0.165 |  |

*Adjusted for age group, sex, diabetes status, cholesterol status, history of heart attack/stroke, and district. †Change = follow-up minus baseline (negative = reduction). Means are predictive margins from survey-weighted regression. Red P-values: statistically significant (P<0.05).
